# Supplementary material for: The interactome of intact mitochondria by cross-linking mass spectrometry provides evidence for coexisting respiratory supercomplexes
Source: Mol Cell Proteomics. 2017 Dec 8;17(2):216–32. doi: 10.1074/mcp.RA117.000470 (PMC5795388; doi:10.1074/mcp.RA117.000470)
Supplement: Supplemental Data [file supp_RA117.000470_133922_0_supp_23978_fzffwf.zip › spectra_annotation/mito_spectra_annotation/374-1-4-1-10-1.pdf]

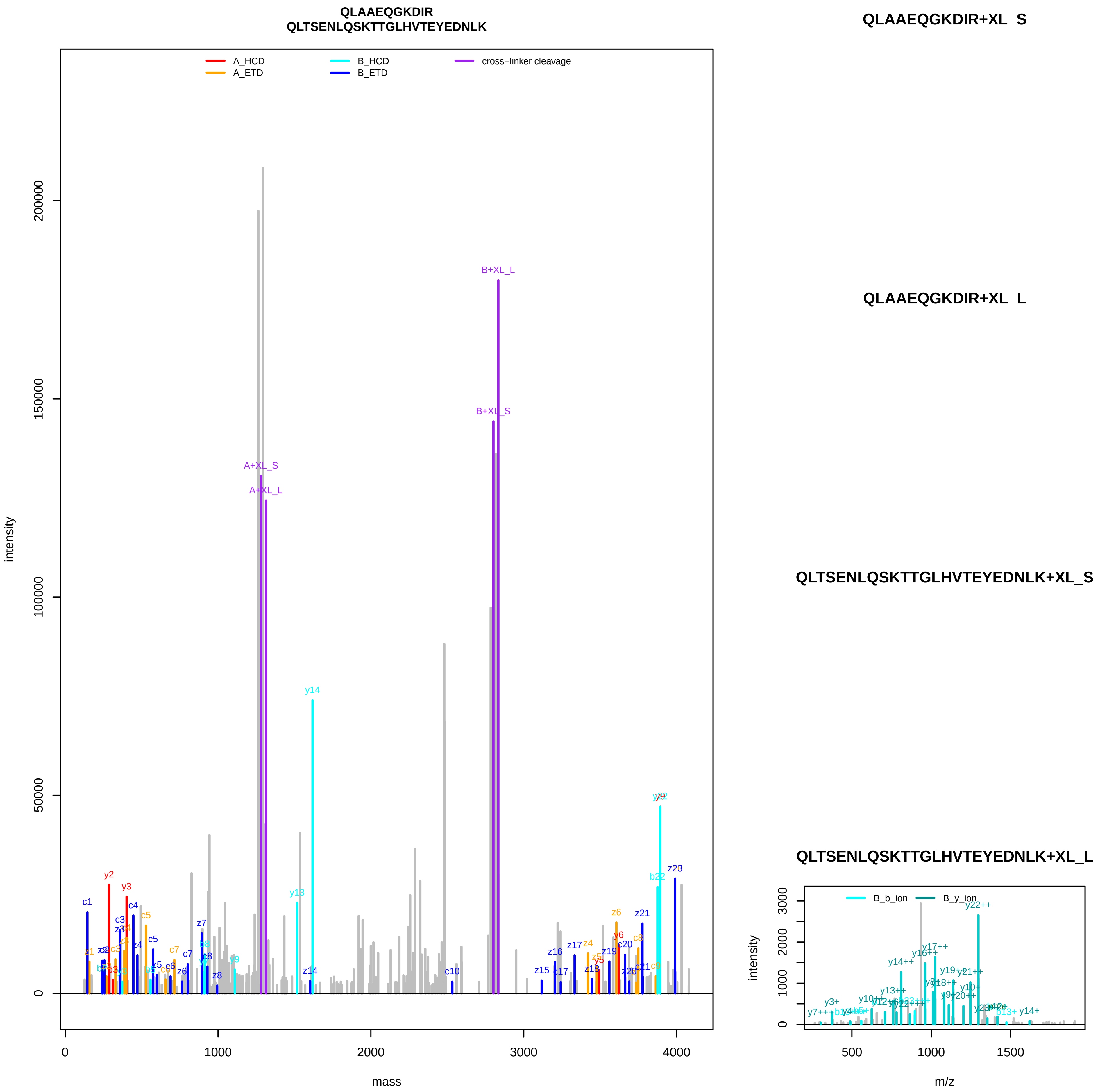

QLAAEQGKDIR
QLTSENLQSKTTGLHVTEYEDNLK
A_HCD
A_ETD
B_HCD
B_ETD
cross−linker cleavage
B+XL_L
B+XL_S
A+XL_S
A+XL_L
y14
y13
y2
y3
c1
c4
c5
z7
z6
c10
z15
z16
c17
z17
z4
z18
z19
c20
z20
z21
c21
z23
b22
c8
c9
y6
y5
z8
c7
intensity
mass
0
50000
100000
150000
200000
1000
2000
3000
4000
QLAAEQGKDIR+XL_S
QLAAEQGKDIR+XL_L
QLTSENLQSKTTGLHVTEYEDNLK+XL_S
QLTSENLQSKTTGLHVTEYEDNLK+XL_L
B_b_ion
B_y_ion
y22++
y17++
y16++
y14++
y19++
y21++
y13++
y18++
y10+
y20++
y3+
y7+++
y14+
b13+
m/z
500
1500
1000
2000
3000
